# Middle Ear Osteoma With Assimilation of Ossicles: An Unusual Case of Conductive Hearing Loss

**DOI:** 10.7759/cureus.38478

**Published:** 2023-05-03

**Authors:** William Crohan, Jafri Kuthubutheen

**Affiliations:** 1 Otolaryngology - Head and Neck Surgery, University of Western Australia, Perth, AUS; 2 Otology, University of Western Australia, Perth, AUS

**Keywords:** osteoma, middle ear tumors, middle ear disease, benign tumor of middle ear, conductive hearing loss

## Abstract

We present a case report of a middle ear osteoma presenting as gradual unilateral conductive hearing loss in a healthy 32-year-old lady. The decision for treatment was influenced by the relatively small burden of the disease, and the size and location of the osteoma, which made the decision for surgical excision prohibitively difficult.

Taking patient wishes and circumstances into account, the decision was made for a bone conduction hearing implant in conjunction with close follow-up.

## Introduction

Middle ear osteoma is a benign tumor that rarely is seen in daily practice. Although its location within the middle ear tends to dictate clinical presentation, it most commonly presents as a slow, progressive form of conductive hearing loss. It is typically treated with surgical excision and ossicular chain reconstruction.

Our case represents a particularly extreme example that involved the assimilation of all ossicles with severe hearing loss. Ultimately, the patient was treated with a conservative treatment strategy and consequently has had an excellent outcome.

This case demonstrates an unusual case of middle ear osteoma and highlights how the decision for treatment should be tailored to each individual based on their unique circumstances.

## Case presentation

The patient is a 28-year-old lady who was referred to the Otology clinic at a major tertiary center with gradual hearing loss in her right ear. She described no history of otalgia, otorrhea, vertigo, facial muscle paralysis, or tinnitus. She had no prior history of ear surgery, nor a documented hearing test prior to presentation. Her past medical history was otherwise notable for recurrent acute otitis media as a child, polycystic ovarian syndrome, obesity, hypothyroidism, depression, and asthma. She regularly took thyroxine, escitalopram, a fluticasone/formoterol combination inhaler, and a salbutamol inhaler as required. She had a family history of ear disease, with her sister requiring grommets as a child. She was not currently working, had never smoked, and was a non-drinker. There was no history of head trauma, noise exposure, or familial hearing loss.

**Figure 1:**
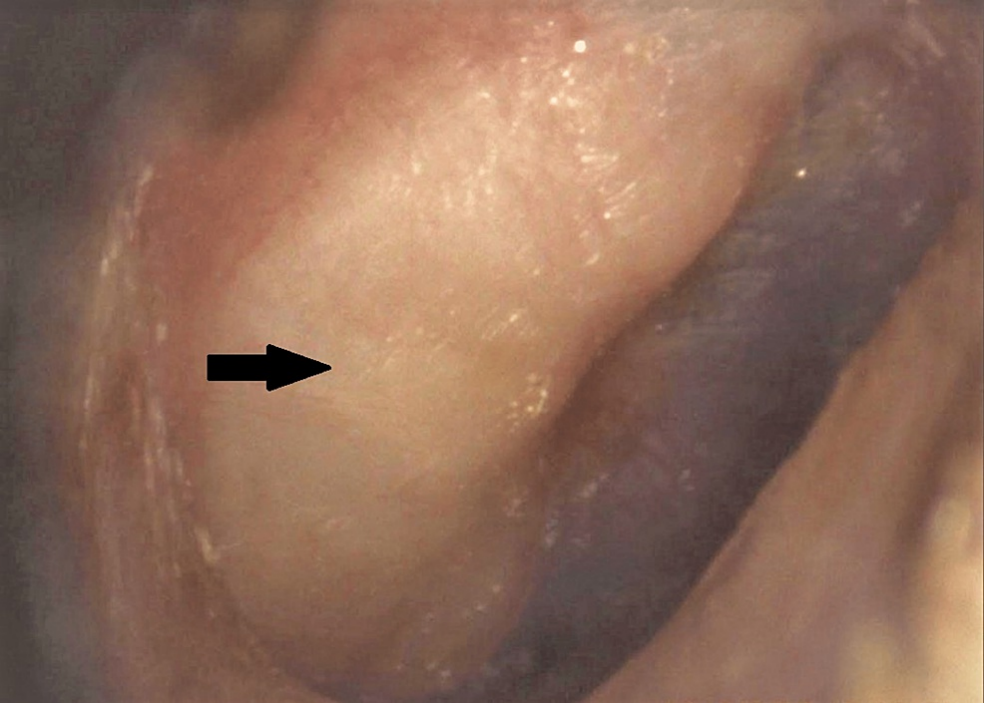
Otoscopy of the right ear demonstrating a whiteish posterior mass or opacification, as highlighted by the arrow.

On examination, there was a clearly defined white opacification of the right tympanic membrane (TM) (Figure 1). It was not clear on examination whether this representation was an opacification of the TM itself or a mass contained medially to the TM. The anterior TM was mobile on pneumotoscopy. Examination of the external acoustic meatus was otherwise unremarkable and there were no other salient features on examination of the left ear, oral cavity, oropharynx, or nasendoscopy of the post-nasal space. Vestibular and head and neck examinations were clinically unremarkable. She tested negatively on Henneberg Test.

Audiology demonstrated severe to moderate conductive hearing loss in the right ear. Pure tone hearing thresholds were 70 decibels (dB) at 500 hertz (Hz), 50 dB at 1000 Hz, and 50 dB at 2000 Hz. There was a Carhart notch on testing of bone conduction hearing thresholds, with 20 dB at 500 Hz, 15 dB at 1000 Hz, and 35 dB at 2000 Hz. Hearing thresholds were unremarkable in the left ear. The right ear had a type B tympanogram, and the left ear had a type A tympanogram. Acoustic reflex testing was not performed.

**Figure 2:**
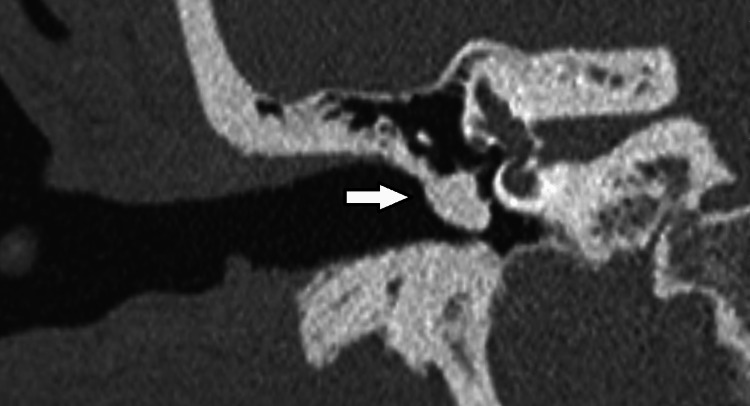
Coronal view of the right ear on CT imaging. Arrow highlights an opacification enveloping handle of the malleus, long process of the incus, and head of the stapes.

**Figure 3:**
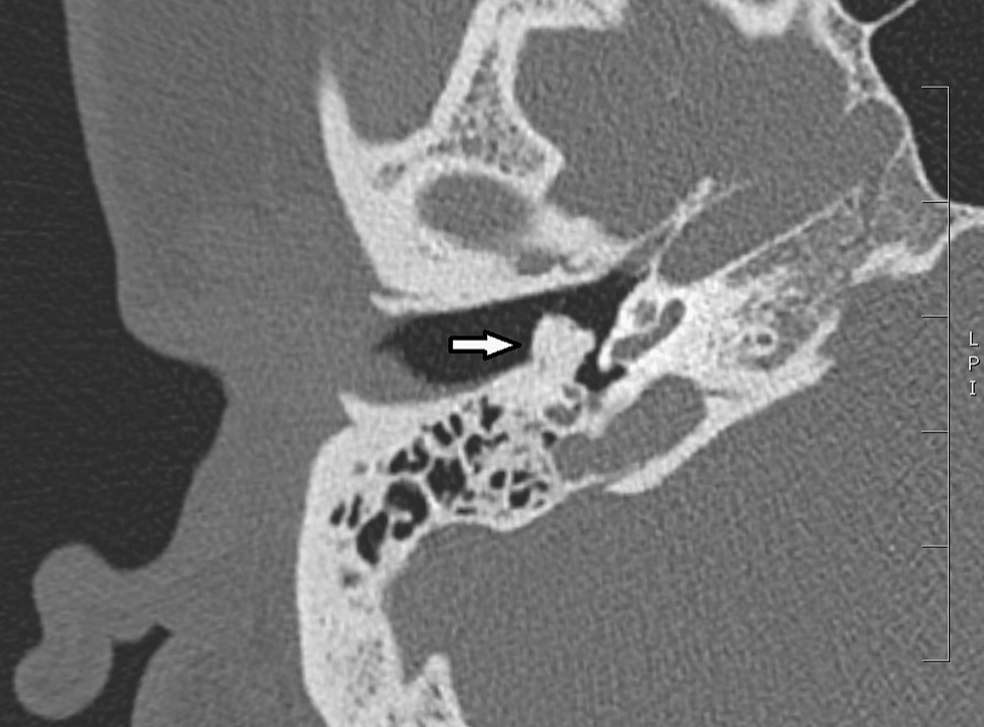
Axial view of right ear on CT imaging, with an arrow highlighting opacification arising from retrotympanum, lateral to aditus.

A computerized tomography (CT) scan of the temporal bones was requested to evaluate the cause of her presentation (Figures 2- 3).

Imaging demonstrated a hyperdense, homogenous mass of 5.7 x 5.4 x 5.2 mm, which was of similar density to surrounding cortical bone at 1500 Hounsfield Units, suggestive of a middle ear osteoma. The mass arose from the posterior wall of the mesotympanum with the posterior half of the pars tensa overlying its lateral margin as supported by clinical examination. It had assimilated the handle of the malleus, the long process of the incus, and the head and neck of the stapes. This was considered to be the likely explanation for her conductive hearing loss. There was a normal appearance of the facial canal, and inner ear structures were morphologically normal.

The patient was referred to the audiology implant clinic for a workup of the possible hearing implant. Aided testing was conducted with a bone-anchored hearing aid on a soft band. In a free field with her left ear masked, she performed well on consonant nucleus consonant testing with a score of 93%.

Subsequent discussion at an implant multidisciplinary team meeting, taking into consideration of expressed patient concerns, led to the decision for a bone conduction implant. It was felt that surgery to remove the osteoma would have posed an unnecessary risk to both the facial nerve and inner ear.

The patient received a Bonebridge implant (Med-El. Innsbruck, Austria) without issue and has had an excellent result. She is very happy with the outcome and is currently under clinical surveillance.

## Discussion

Temporal bone osteomas are benign tumors typically found in the external auditory canal. Their occurrence within the middle ear is rare, though not unprecedented, with 39 cases documented since 1964 [1-6]. The etiology of middle ear osteomas is unclear. The first documented case report of middle ear osteoma described a case involving siblings, suggesting a possible genetic predisposition toward their development [1]. Contemporary case reports have been more suggestive of a predominantly inflammatory origin [7,8]. This appears to be an ongoing area of debate. Their location in the middle ear tends to dictate clinical presentation, which is typically a history of slow, progressive conductive hearing loss [8]. Occasionally the osteoma is visible through the TM and can be mistaken for a congenital cholesteatoma [4]. As a point of interest, several studies have noted a Carhart notch, which can lead to an incorrect diagnosis of otosclerosis if imaging is not performed [6,8,9].

Middle ear osteoma has been shown to most commonly arise from the promontory [8]. While there have been several instances of an osteoma assimilating the malleus or incus, there have only been four cases involving the stapes [3,6,8,10,11], and there has never been a case before assimilating all three ossicles. To our knowledge, this is the first case reported to have assimilated all three ossicles.

Most reported cases have been treated with surgical excision of the osteoma, with respect to their location and structures involved [8]. Given that osteomas tend to grow very slowly, certain centers have advocated for close monitoring instead of surgery, particularly if invasive management would potentially compromise the inner ear and facial nerve [6,12,13]. In this instance, it was felt that the optimal surgical approach would be to perform a mastoidectomy for full visualization, with a stapedectomy to disarticulate the osteoma from the stapes footplate. A primary or secondary ossicular chain reconstruction could then be considered. Given the risks inherent to such a procedure, and the relatively small burden of disease associated with the osteoma, it was agreed that the patient should undergo assessment for a bone conduction implant, with ongoing surveillance of the osteoma.

To our knowledge, no patient has received a bone conduction implant as part of their management, though this option has been previously discussed [6]. Bone conduction implants may consequently represent a new, pragmatic treatment option for this condition.

## Conclusions

Middle ear osteoma is a rare cause of conductive hearing loss. We present a particularly extreme example involving the assimilation of all ossicles of the middle ear. To our knowledge, this has not been reported in the literature previously. The patient was treated conservatively following a multidisciplinary discussion between audiologists, surgeons, and the patient. To our knowledge, this is the first time a bone conduction hearing aid with close follow-up has been used to treat or compensate for a middle ear osteoma.
